# Modulation of Endothelial Injury Biomarkers by Traditional Chinese Medicine LC in Systemic Lupus Erythematosus Patients Receiving Standard Treatments

**DOI:** 10.1038/srep19622

**Published:** 2016-02-05

**Authors:** Hen-Hong Chang, Shue-Fen Luo, Yin-Tzu Hsue, Ching-Mao Chang, Tzung-Yan Lee, Yu-Chuen Huang, Ming-Ling Hsu, Yu-Jen Chen

**Affiliations:** 1 Research Center for Chinese Medicine and Acupuncture, and School of Chinese Medicine, China Medical University, Taichung, Taiwan; 2 Division of Rheumatology, Allergy and Immunology, Chang Gung Memorial Hospital Linkou branch, Taoyuan, Taiwan; 3 Division of Rheumatology, Allergy and Immunology, Department of Internal Medicine, Changhua Christian Hospital, Changhua, Taiwan; 4 Center for Traditional Medicine, Taipei Veterans General Hospital, Taipei, Taiwan; 5 School of Traditional Chinese Medicine, College of Medicine, Chang Gung University, Taoyuan, Taiwan; 6 Department of Medical Research, China Medial University Hospital, Taichung, Taiwan; 7 School of Chinese Medicine, College of Chinese Medicine, China Medical University, Taichung, Taiwan; 8 Department of Medical Research, MacKay Memorial Hospital, Taipei, Taiwan; 9 Department of Radiation Oncology, MacKay Memorial Hospital, Taipei, Taiwan

## Abstract

LC is an herbal remedy effectively reduced therapeutic dosage of glucocorticoid for systemic lupus erythematosus (SLE) patients in clinical trial (ISRCTN81818883). This translational research examined the impact of LC on biomarkers of endothelial injury in the enrolled subjects. Fifty seven patients with SLE were randomized to receive standard treatment without or with LC supplements. Blood samples were taken serially for quantification of endothelial progenitor cells (EPCs), circulating endothelial cells (CECs) and serological factors. The proportion of EPCs in the placebo group continued to increase during trial and was further elevated after withdrawal of standard treatment. The EPC ratio of LC group remained stationary during the entire observation period. The CEC ratio in placebo group exhibited an increasing trend whereas that in LC group declined. The ratio of apoptotic CECs had an increasing trend in both groups, to a lesser extent in LC group. After treatment, the levels of VEGF and IL-18 have a trend declined to a level lower in the LC group than the placebo group. No significant alteration was noted in serum levels of IFN-α, IL-1β and IL-6. The reduction of the steroid dosage by adding LC might be correlated with less extensive endothelial injury in SLE patients.

The increase in cardiovascular risk of morbidity and mortality has been observed in patients with autoimmune diseases, including systemic lupus erythematosus (SLE)^1^. It has been proposed that systemic inflammation and autoimmune-related reactions play a pivotal role in the increase of cardiovascular risk. Endothelial damage and dysfunction mediated by the production of adhesion molecules and the overexpression of pro-inflammatory cytokines is regarded as an important consequence of autoimmune-related reactions^2^,^3^. Given that traditional Framingham risk factors^1^ cannot represent the accelerated development of atherosclerosis in SLE patients^4^, novel biomarkers for endothelial injury that proceed from clinical cardiovascular diseases are needed.

The detachment of endothelial cells from injured endothelium into circulation (circulating endothelial cell, CECs)^5^ and the mobilization of endothelial progenitor cells (EPCs) from bone marrow^6^ have been developed as biomarkers for vascular endothelial damage, the primary event in atherosclerosis. EPCs express endothelial [vascular endothelial growth factor (VEGF) receptor-2] and haematopoietic (CD34 and CD133) cell markers. During differentiation to mature EPCs, CD133 expression is lost and begins to express vascular endothelial (VE)-cadherin and von Willebrand factor^7^. The level of circulating EPCs has been shown to be inversely correlated with cardiovascular risk^8^. The levels of CECs and the amount of apoptotic CECs has been reported as representing the extent of endothelial damage^9^,^10^.

Pro-angiogenic factors, such as VEGF, can mobilize EPCs and recruit EPCs to the injured endothelial lesion^11^. By contrast, anti-angiogenic factors exert an opposite effect. Several pro-inflammatory and immunosupressor cytokines, such as Interleukin-1β (IL-1β) 12, IL-6^13^, IL-17^14^, TGF-β^15^, and interferon-α (IFN-α)^16^,^17^, are involved in EPC mobilization, recruitment, proliferation and function. Most of these cytokines have been reported to be deregulated in autoimmune patients and may involve immune-mediated mechanisms in vascular damage.

In the present work, we measured the amounts of circulating EPCs, CECs and apoptotic CECs in SLE patients in a clinical trial that evaluated the effect of adding a Chinese medicinal herb to the standard therapy. We also evaluated the possible correlations between treatment outcome and serum levels of cytokines relevant for SLE.

## Methods

### Patients and Blood Sampling

The study protocol was approved by the Institutional Review Board and Ethics Committee of Chang Gung Memorial Hospital, Linkou, Taiwan. The study was performed in accordance with the approved guidelines. The informed consent was obtained from all subjects. The clinical trial was registered with number ISRCTN81818883 from November 2007 to October 2010, 85 SLE patients were screened via rheumatology clinics, and 62 of them were enrolled into the trial; 31 patients were randomized into a placebo group and another 31 patients into an LC group. The LC formula was designed to combine the contents of an L (Long Dan Xie Gan Tang) and C (Zhi Bai Di Huang Wan) regimen. LC was given orally for 16 weeks and withdrawn for 8 weeks, and the evaluations were performed every 4 weeks. Adequate peripheral blood samples with satisfied quality for assessment were obtained from 28 patients in the placebo group and 29 in the LC group every 4 weeks before, during and after treatment. These blood samples were subjected to measurement of serum circulating EPCs, CECs, apoptotic CECs and soluble factors, including IFN-α, IL-1β, IL-6, IL-18 and VEGF.

### Flow Cytometry

To measure circulating EPCs and CECs, a method from Duda *et al*.^18^ was adapted with modifications as in our previous study^19^. Flow cytometry was used for whole blood analysis without enrichment procedures to avoid manipulation artifacts. The EPCs were characterized as CD31^+^ VEGFR-2^+^ CD45^dim^ CD133^+^; mature CECs were defined as CD31^Bright^ VEGFR-2^+^ CD45^-^ CD133^-^. The chromophores conjugated with specific antibodies used in this study included CD31-FITC (BD Pharmingen, San Diego, CA), CD133-PE (Miltenyi Biotec, Auburn, CA), CD45-PerCP (BD Pharmingen), and VEGFR2-PE (R&D Systems, Minneapolis, MN). For apoptotic CECs, AAD-7 was also applied to stain the apoptotic CECs. During the analysis of flow cytometry data, the mononuclear cell population was gated to avoid RBC, platelet, cell debris, and neutrophil contamination; 100,000 events in the gated population were collected by a FACScaliber flow cytometer (BD Biosciences, San Jose, CA, USA). The acquisition data were collected and analyzed using CellQuest Software (BD Biosciences).

### Measurement of serum cytokines

Serum levels of proinflammatory and angiogenic factors were quantified by a sandwich enzyme-linked immunosorbent assay (ELISA). VEGF was measured using the Duoset ELISA kits from R&D Systems (Wiesbaden, Germany). IFN-α, IL-1β, IL-6, and IL-18 were measured using Quantikine ELISA kits (R&D Systems) according to the manufacturer’s instructions.

### Statistical analysis

Statistical analyses were performed using the software package SPSS 18 (SPSS, Chicago, IL). Linear mixed model was applied to compare the disease activity of SLE (SLEDAI score) score, circulating endothelial cell and blood biochemistry between groups. *p* < 0.05 was considered significant.

## Results

### Characteristics of patients

Among enrolled patients, the clinical parameters showed a higher disease activity in LC group in terms of the SLEDAI score, C3, C4 and anti-dsDNA antibody before treatment (unpublished data). The significant reduction in the administered dosage of steroids was noted in the LC group (unpublished data).

### Dynamic changes in CECs, EPCs and apoptotic CECs

**Figure 1:**
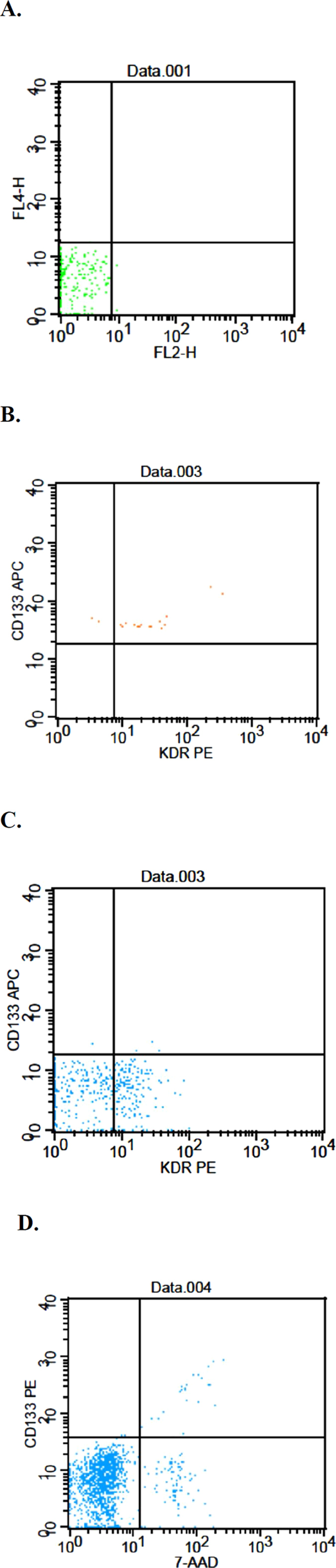
Representative data for flow cytometric analysis of EPC, CEC and apoptotic CEC. (**A**) Dot plot shows the gating strategy used to exclude debris; (**B**) The EPCs were characterized as CD31^+^ VEGFR-2^+^ CD45^dim^ CD133^+^; (**C**) The CECs were defined as CD31^Bright^ VEGFR-2^+^ CD45^−^ CD133^−^; (**D**) AAD-7 staining was used for identification and quantification of apoptotic CECs.

**Figure 2:**
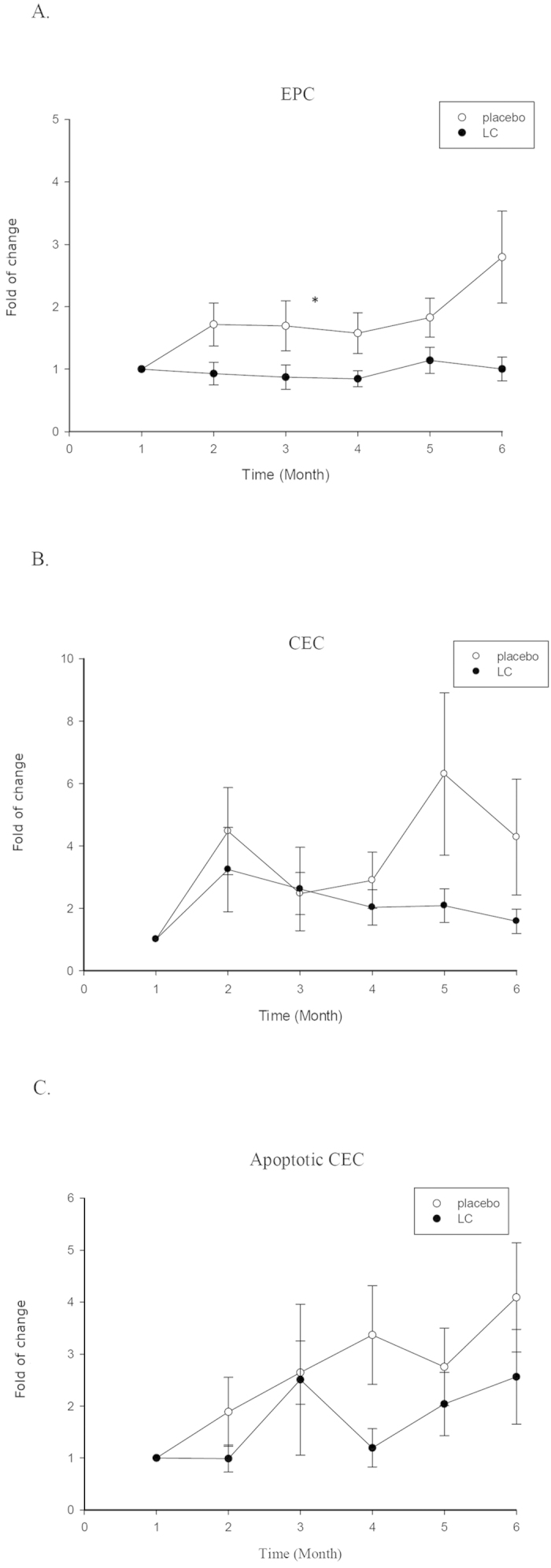
Serial changes in the amounts of EPCs, CECs and apoptotic CECs. (**A**) EPC; (**B**) CEC; (**C**) apoptotic CEC. The ratios of EPC, CEC and apoptotic CEC were calculated by comparing pretreatment amounts of these cell populations of each own subject. *p <0.05.

To examine the correlation between clinical outcomes and serological markers, the quantities of serum cytokines, EPCs, CECs and apoptotic CECs were serially estimated. The representative result of flow cytometric analysis for EPCs, CECs and apoptotic CECs was demonstrated in Fig. 1. As shown in Fig. 2A, the proportion of EPCs at pretreatment levels in the placebo group increased from the beginning to the end of trial (*p* < 0.05). It was further elevated 8 weeks after withdrawal of the standard treatment. Intriguingly, the EPCs ratio of LC group remained stationary during the entire observation period, including that after withdrawal of standard treatment. The CEC ratio in placebo group exhibited an increasing trend (not significantly different, *p* = 0.172) with marked elevation at weeks 4 and 16 during the standard treatment. The final CEC ratio at week 24 (8 weeks after treatment) in the placebo group remained higher than the pretreatment ratio. In the LC group, the CEC ratio was elevated at week 4 followed by a continuous decline to the end, close to the pre-treatment ratio. The apoptotic CEC ratio had an increasing trend (not significantly different, *p* = 0.211) in both groups, but the extent in the LC group was less than the placebo group.

### Profiles of serum cytokine levels

**Figure 3:**
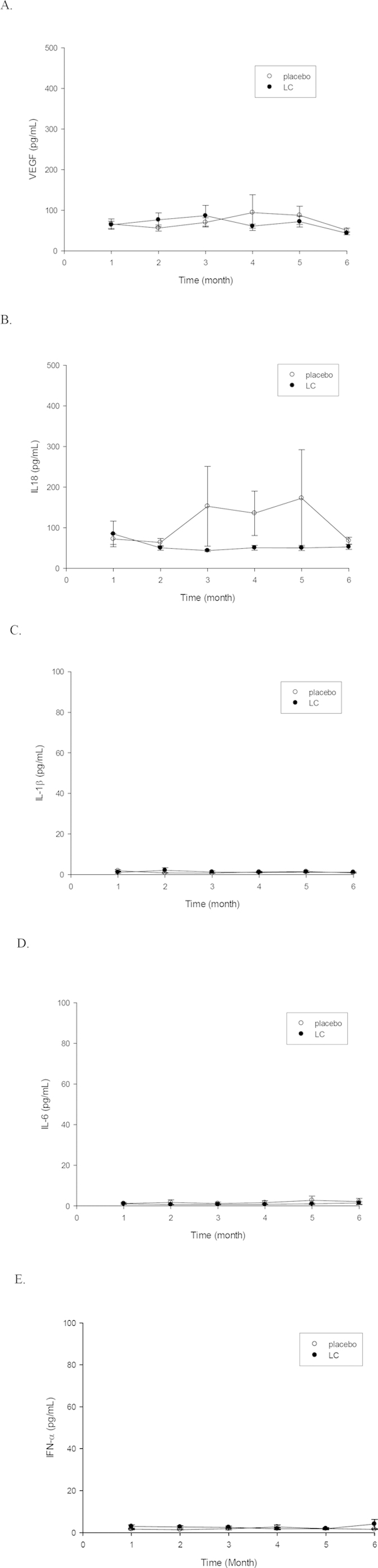
Serum levels of cytokines in SLE patients. (**A**) VEGF; (**B**) IL-18; (**C**) IL-1β; (**D**) IL-6; E, IFN-α.

We next examined the serial changes in serum levels of IL-18, VEGF, IFN-α, IL-1β, and IL-6. Before beginning treatment, the serum levels of IL-18 were higher in the LC group, which was compatible with clinical SLEDAI scores for disease activity. After treatment, the IL-18 levels of the placebo and LC groups became similar at the 2^nd^ month of standard treatment and, furthermore, the levels of IL-18 declined to levels lower than the placebo group (not significantly different, p  = 0.190). Serum levels of VEGF, exhibited a similar profile as IL-18. No significant alteration was noted in serum levels of IL-18, VEGF, IFN-α, IL-1β and IL-6 (Fig. 3).

## Discussion

In SLE patients receiving the standard treatment, we found that levels of endothelial injury biomarkers incremented during the standard treatment and these markers, as well as serum levels of VEGF and IL-18, could be suppressed by a combinatory treatment with Chinese medicinal herb LC.

Disease-modifying antirheumatic drugs (DMARDs) are used as a prevention to reduce the incidence of flares from the disease and lower the need for steroid use. Antimalarial agents, such as hydroxychloroquine^20^ and immunosuppressants are commonly used as DMARDs. DMARDs are regarded as a part of the standard treatments for autoimmune disease. Their use during pregnancy can prevent flares of the disease compromising the health of the mother and fetus^21^. Our results from a randomized placebo-controlled clinical trial show that LC could reduce the needed dose of steroid during standard treatment of SLE (unpublished data). Thus, LC might have the potential to be developed as a novel DMARD. The biomarker data of the present study further provides a correlation between endothelial injury and the effect of LC on SLE patients.

It has been demonstrated that autoimmune reactions enhanced the release of bone marrow progenitor cells, which include EPCs, in patients with SLE^14^. The number of circulating EPCs in active SLE patients was not significantly different from healthy controls, but their functions were partly impaired, including proliferation, adhesion, migration, and tube formation^22^. EPCs are a heterogeneous population whose physiological role is to carry out angiogenesis and vascular repair^23^. Because of their potential role in vascular repair, the amount of circulating EPCs is considered a surrogate marker for vascular dysfunction and cardiovascular risk, and thus a promising tool in cell therapy for cardiovascular diseases, especially in connective tissue diseases.

In addition to changes in cellular profiles, several cytokines, such as proinflammatory cytokines and VEGF, involve vascular damage and angiogenesis. We demonstrated that elevated VEGF and IL-18 levels, but not IFN-α, IL-1β or IL-6 levels, were suppressed by the LC treatment in SLE patients. Serum levels of IL-18, an inflammosome activator, were shown to be elevated in SLE and correlated with EPC/circulating angiogenic cell (CACs) dysfunction. In *ex vivo* cultures of EPCs/CACs from SLE patients, exogenous IL-18 inhibited endothelial differentiation and neutralization of IL-18 could restore their differentiation into endothelial cells^24^. It suggests a correlation between the biological effects of IL-18 and the impaired vascular repair *in vivo*. Our clinical results showing that LC treatments reduced the needed steroid dosage and suppressed the level of IL-18 may provide a line of evidence for this correlation.

The current development of biological markers for vascular damage and angiogenesis in SLE are mainly from investigations focusing on the correlation between these biomarkers, disease activity and related cardiovascular complications^25^. Given EPCs mobilized from bone marrow may facilitate non-physiological angiogenesis and resultant unfavorable vascular events; EPCs could be regarded as treatment targets for SLE. On the other hand, our findings that LC treatments reduced the amount of EPCs during standard steroid therapy may imply that the effect of LC is to target EPCs. Surely, this assumption needs further validation by both *in vitro* and *in vivo* experiments. By current data, we may have no adequate evidence to differentially suggest the alterations in biomarkers of endothelial injury are direct effects of LC or a steroid-sparing effect.

In conclusion, the levels of endothelial injury biomarkers, as well as VEGF and IL-18, in SLE patients during standard treatment could be suppressed by a combinatory treatment with the traditional Chinese medicine LC.

## Additional Information

**How to cite this article**: Chang, H.-H. *et al*. Modulation of Endothelial Injury Biomarkers by Traditional Chinese Medicine LC in Systemic Lupus Erythematosus Patients Receiving Standard Treatments. *Sci. Rep*. **6**, 19622; doi: 10.1038/srep19622 (2016).

## References

[b1] Manzi S. . Age-specific incidence rates of myocardial infarction and angina in women with systemic lupus erythematosus: comparison with the Framingham Study. Am J Epidemiol 145, 408–415 (1997). 9048514 10.1093/oxfordjournals.aje.a009122

[b2] Bauer J. W. . Elevated serum levels of interferon-regulated chemokines are biomarkers for active human systemic lupus erythematosus. PLoS Med 3, e491 (2006). 17177599 10.1371/journal.pmed.0030491 PMC1702557

[b3] Bauer J. W. . Interferon-regulated chemokines as biomarkers of systemic lupus erythematosus disease activity: a validation study. Arthritis Rheum 60, 3098–3107 (2009). 19790071 10.1002/art.24803 PMC2842939

[b4] Sacre K. . Increased arterial stiffness in systemic lupus erythematosus (SLE) patients at low risk for cardiovascular disease: a cross-sectional controlled study. PLoS One 9, e94511 (2014). 24722263 10.1371/journal.pone.0094511 PMC3983200

[b5] Goon P. K., Boos C. J. & Lip G. Y. Circulating endothelial cells: markers of vascular dysfunction. Clin Lab 51, 531–538 (2005). 16285476

[b6] Lin Y., Weisdorf D. J., Solovey A. & Hebbel R. P. Origins of circulating endothelial cells and endothelial outgrowth from blood. J Clin Invest 105, 71–77 (2000). 10619863 10.1172/JCI8071 PMC382587

[b7] Zampetaki A., Kirton J. P. & Xu Q. Vascular repair by endothelial progenitor cells. Cardiovasc Res 78, 413–421 (2008). 18349136 10.1093/cvr/cvn081

[b8] Werner N. . Circulating endothelial progenitor cells and cardiovascular outcomes. N Engl J Med 353, 999–1007 (2005). 16148285 10.1056/NEJMoa043814

[b9] Del Papa N. . Circulating endothelial cells as a marker of ongoing vascular disease in systemic sclerosis. Arthritis Rheum 50, 1296–1304 (2004). 15077314 10.1002/art.20116

[b10] Rajagopalan S. . Endothelial cell apoptosis in systemic lupus erythematosus: a common pathway for abnormal vascular function and thrombosis propensity. Blood 103, 3677–3683 (2004). 14726373 10.1182/blood-2003-09-3198

[b11] Tepper O. M. . Adult vasculogenesis occurs through *in situ* recruitment, proliferation, and tubulization of circulating bone marrow-derived cells. Blood 105, 1068–1077 (2005). 15388583 10.1182/blood-2004-03-1051

[b12] Yang L. . Interleukin-1 beta increases activity of human endothelial progenitor cells: involvement of PI3K-Akt signaling pathway. Inflammation 35, 1242–1250 (2012). 22371121 10.1007/s10753-012-9434-9

[b13] Fan Y. . Interleukin-6 stimulates circulating blood-derived endothelial progenitor cell angiogenesis *in vitro*. J Cereb Blood Flow Metab 28, 90–98 (2008). 17519976 10.1038/sj.jcbfm.9600509 PMC2581498

[b14] Rodriguez-Carrio J. . Circulating endothelial cells and their progenitors in systemic lupus erythematosus and early rheumatoid arthritis patients. Rheumatology (Oxford) 51, 1775–1784 (2012). 22753774 10.1093/rheumatology/kes152

[b15] Sales V. L. . Transforming growth factor-beta1 modulates extracellular matrix production, proliferation, and apoptosis of endothelial progenitor cells in tissue-engineering scaffolds. Circulation 114, I193–199 (2006). 16820571 10.1161/CIRCULATIONAHA.105.001628

[b16] Thacker S. G. . The detrimental effects of IFN-alpha on vasculogenesis in lupus are mediated by repression of IL-1 pathways: potential role in atherogenesis and renal vascular rarefaction. J Immunol 185, 4457–4469 (2010). 20805419 10.4049/jimmunol.1001782 PMC2978924

[b17] Denny M. F. . Interferon-alpha promotes abnormal vasculogenesis in lupus: a potential pathway for premature atherosclerosis. Blood 110, 2907–2915 (2007). 17638846 10.1182/blood-2007-05-089086 PMC2018671

[b18] Duda D. G., Cohen K. S., Scadden D. T. & Jain R. K. A protocol for phenotypic detection and enumeration of circulating endothelial cells and circulating progenitor cells in human blood. Nat Protoc 2, 805–810 (2007). 17446880 10.1038/nprot.2007.111 PMC2686125

[b19] Lin C. C. . Profiles of circulating endothelial cells and serum cytokines during adjuvant chemoradiation in rectal cancer patients. Clin Transl Oncol 15, 855–860 (2013). 23401019 10.1007/s12094-013-1004-6

[b20] Ostensen M. Treatment with immunosuppressive and disease modifying drugs during pregnancy and lactation. Am J Reprod Immunol 28, 148–152 (1992). 1285866 10.1111/j.1600-0897.1992.tb00778.x

[b21] Ostensen M. & Forger F. Management of RA medications in pregnant patients. Nat Rev Rheumatol 5, 382–390 (2009). 19506586 10.1038/nrrheum.2009.103

[b22] Deng X. L., Li X. X., Liu X. Y., Sun L. & Liu R. Comparative study on circulating endothelial progenitor cells in systemic lupus erythematosus patients at active stage. Rheumatol Int 30, 1429–1436 (2010). 19847436 10.1007/s00296-009-1156-4

[b23] Dome B. . Circulating bone marrow-derived endothelial progenitor cells: characterization, mobilization, and therapeutic considerations in malignant disease. Cytometry A 73, 186–193 (2008). 18000872 10.1002/cyto.a.20480

[b24] Kahlenberg J. M. . Inflammasome activation of IL-18 results in endothelial progenitor cell dysfunction in systemic lupus erythematosus. J Immunol 187, 6143–6156 (2011). 22058412 10.4049/jimmunol.1101284 PMC3221936

[b25] Mak A. & Kow N. Y. Imbalance between endothelial damage and repair: a gateway to cardiovascular disease in systemic lupus erythematosus. Biomed Res Int 2014, 178721 (2014). 24790989 10.1155/2014/178721 PMC3984775

